# Unintentional falls mortality in China, 2006-2016

**DOI:** 10.7189/jogh.09.010603

**Published:** 2019-06

**Authors:** Peixia Cheng, Lijun Wang, Peishan Ning, Peng Yin, David C Schwebel, Jiangmei Liu, Jinlei Qi, Guoqing Hu, Maigeng Zhou

**Affiliations:** 1 Department of Epidemiology and Health Statistics, Xiangya School of Public Health, Central South University. Changsha, China; 2 National Center for Chronic and Noncommunicable Disease Control and Prevention, Chinese Center for Disease Control and Prevention. Beijing, China; 3 Department of Psychology, University of Alabama at Birmingham, Birmingham, Alabama, USA; * Joint first authors; # Joint senior authors

## Abstract

**Background:**

To examine trends in unintentional falls mortality from 2006 to 2016 in China by location (urban/rural), sex, age group and mechanism.

**Methods:**

Mortality data were retrieved from the National Disease Surveillance Points system (DSPs) of China, a nationally representative data source. Percent change in mortality between 2006 and 2016 was calculated as “mortality rate ratio - 1” based on a negative binomial regression model.

**Results:**

The crude unintentional falls mortality was 9.55 per 100 000 population in 2016. From 2006 to 2016, the age-adjusted unintentional falls mortality increased by 5% (95% confidence interval (CI) = 1%-9%), rising from 7.65 to 8.03 per 100 000 population. Males, rural residents and older age groups consistently had higher falls mortality rates than females, urban residents and younger age groups. Falls on the same level from slipping, tripping and stumbling (W01) was the most common mechanisms of falls mortality, accounting for 29% of total mortality.

**Conclusions:**

Unintentional falls continued to be a major cause of death in China from 2006 to 2016. Empirically-supported interventions should be implemented to reduce unintentional falls mortality.

Unintentional falls are a leading cause of injury morbidity and mortality globally, placing significant burden on victims, families and societies. The World Health Organization (WHO) estimated approximately 392 000 people died from unintentional falls in 2004 worldwide [1]. Largely through the combined influence of population growth and population ageing, the number of deaths due to falls increased by 21% between 2005 and 2015, [2] and fall-induced disability-adjust life years per 100 000 population increased by 14% between 2006 and 2016 [3]. Falls are predicted to rise to be the 17^th^ leading cause of all deaths by 2030 if preventive efforts are not taken [4]. In China, unintentional falls are the leading cause of fatal and non-fatal injuries for people aged ≥65 years [5,6].

High-quality epidemiological evidence offers a base to assess the severity of a public health problem, develop interventions, and then evaluate the effectiveness of interventions. In China, the circumstance and trends for falls mortality has been reported in some scattered epidemiological studies [7-9], most of which focus on specific segments of the population and/or in limited geographical areas with small sample sizes, or they only report overall falls rates on sex- and age-based trends over time but lack more detailed data by mechanism (eg, Global Burden of Disease (GBD) estimates [10]; Chinese Health Statistics Yearbook [11]).

The primary objective of this study, therefore, was to examine the circumstances and trends of unintentional falls mortality in China by location (urban/rural), sex, age group, and mechanism from 2006 to 2016. We used data from the National Disease Surveillance Points system (DSPs) [12] to conduct our analyses.

## METHODS

### Data source

Mortality data were retrieved from the DSPs, a nationally representative death registration system that was expanded between 2004 and 2006 to include 161 surveillance points (64 urban, 97 rural) across all 31 Chinese provinces [13,14]. Each surveillance point represents a single district (urban area) or county (rural area); all deaths that occur at the surveillance points are collected and the causes of death are coded using ICD-10 codes by trained coders in hospitals or local CDC offices. A standardized and validated web-based approach has been used to report death cases in the DSPs since 2008 [15]. The DSPs surveillance points were expanded again in 2013, from 161 to 605 [14], but to eliminate bias from the expansion of surveillance points, in this study we limited analysis to mortality data from the same 161 surveillance points reporting data consistently from 2006 to 2016. Details of the DSPs methodology are reported in previous publications [16].

### Classification of unintentional falls

The International Classification of Diseases-10th Revision (ICD-10) organizes unintentional falls into 20 subcategories (W00-W19) [17]. Based on preliminary analyses, we combined categories with extremely small death rates to present unintentional falls as seven categories: (1) FSLS: falls on same level from slipping, tripping and stumbling (W01); (2) FRF: furniture related falls (W06-W08); (3) OFSL: other falls on same level (W00, W02-W05, W09 and W18); (4) FSS: falls on and from stairs and steps (W10); (5) FBS: falls from, out of or through a building or structure (W13); (6) OFOL: other falls from one level to another (W11, W12 and W14-W17); and (7) UF: unspecified falls (W19).

### Demographic variables

Four demographic factors covered by the DSPs were considered in data analyses: location (urban/rural), sex, age group and mechanism. Based on previous research [18] and preliminary analyses (not shown here), age was divided into seven groups: 0-4 years, 5-14 years, 15-24 years, 25-44 years, 45-64 years, 65-74 years, and 75 years and older.

### Statistical analysis

Age-standardized mortality rates were calculated according to the 2010 census population. Population-based mortality rates and 95% confidence intervals (95% CIs) were calculated for each year. Considering the over-dispersion of fall-induced deaths, percent change in mortality between 2006 and 2016 was estimated using negative binomial regression, which was calculated as (mortality rate ratio – 1) × 100%. All statistical analyses were performed using Stata 12.1. A *P* value*<*0.05 was considered statistically significant.

## RESULTS

### Overall unintentional falls mortality

**Figure 1:**
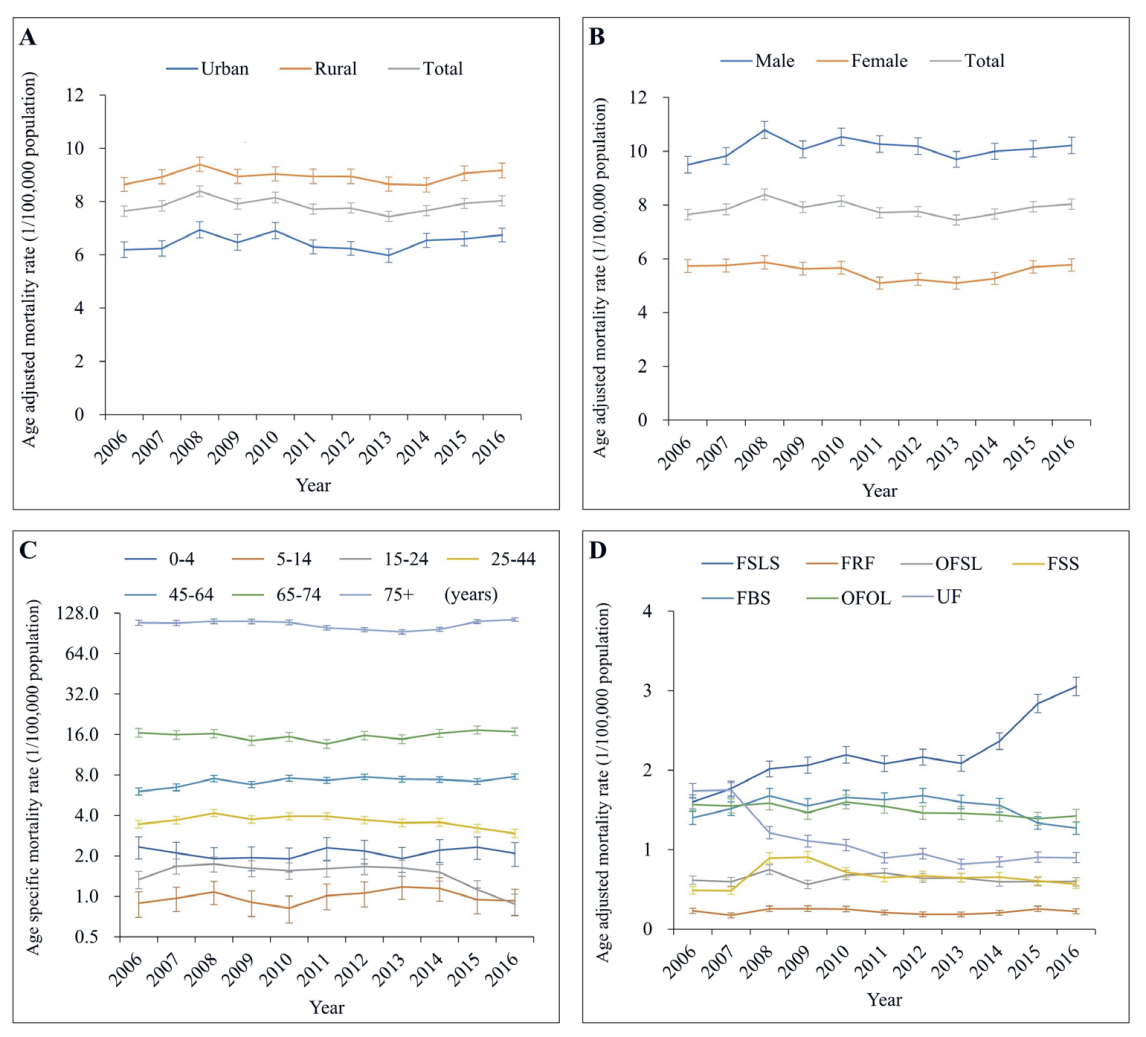
Mortality rates from unintentional falls by location (urban/rural), sex, age group and mechanism in China, 2006-2016. By location (panel **A**), by sex (panel **B**), by age group (panel **C**), by mechanism (panel **D**). Mortality rates in panels A, B and D were age-standardized based on the population of China in 2010. FSLS – falls on same level from slipping, tripping and stumbling (W01), FRF – furniture related falls (W06-W08), OFSL – other falls on same level (W00, W02-W05, W09 and W18), FSS – falls on and from stairs and steps (W10), FBS – falls from, out of or through building or structure (W13), OFOL – other falls from one level to another (W11, W12 and W14-W17), UF – unspecified falls (W19).

From 2006 to 2016, a total of 69 863 unintentional fall-induced deaths were captured by the DSPs. In general, the overall age-adjusted unintentional falls mortality remained roughly stable between 2006 and 2013, with a small fluctuation from 2007 to 2009, and then increased gradually after 2013 (**Figure 1**). Between 2006 and 2016, age-standardized mortality from unintentional falls increased 5% (95% confidence interval CI: 1%-9%), changing from 7.65 to 8.03 per 100 000 population. The crude mortality from unintentional falls was 9.55 per 100 000 population in 2016 (**Table 1**).

**Table 1:** Unintentional falls mortality per 100 000 population (standard error) by age group and mechanism in China, 2006-2016*

Variable	2006	2007	2008	2009	2010	2011	2012	2013	2014	2015	2016	% change in rate (95% CI)†
	7.65 (0.10)	7.84 (0.10)	8.39 (0.10)	7.92 (0.10)	8.15 (0.10)	7.72 (0.10)	7.76 (0.10)	7.44 (0.09)	7.67 (0.10)	7.93 (0.10)	8.03 (0.10)	5 (1 to 9)
**Location:**												
Urban	6.19 (0.15)	6.24 (0.15)	6.94 (0.15)	6.47 (0.15)	6.91 (0.15)	6.30 (0.13)	6.24 (0.13)	5.97 (0.13)	6.55 (0.13)	6.60 (0.13)	6.75 (0.13)	9 (3 to 16)
Rural	8.64 (0.13)	8.93 (0.14)	9.40 (0.14)	8.95 (0.14)	9.04 (0.14)	8.95 (0.14)	8.95 (0.14)	8.66 (0.14)	8.63 (0.14)	9.07 (0.14)	9.17 (0.14)	6 (2 to 11)
**Sex:**												
Male	9.50 (0.16)	9.82 (0.16)	10.79 (0.17)	10.07 (0.16)	10.54 (0.16)	10.27 (0.16)	10.19 (0.16)	9.70 (0.15)	10.00 (0.15)	10.09 (0.16)	10.22 (0.15)	8 (3 to 12)
Female	5.73 (0.12)	5.75 (0.12)	5.87 (0.12)	5.63 (0.12)	5.66 (0.12)	5.10 (0.11)	5.23 (0.11)	5.10 (0.11)	5.27 (0.11)	5.70 (0.12)	5.77 (0.12)	1 (-5 to 7)
**Age group (years):**												
0-4	2.33 (0.22)	2.10 (0.21)	1.91 (0.20)	1.94 (0.20)	1.90 (0.20)	2.30 (0.23)	2.17 (0.22)	1.91 (0.21)	2.21 (0.22)	2.32 (0.23)	2.09 (0.21)	-10 (-32 to 18)
5-14	0.89 (0.10)	0.97 (0.10)	1.08 (0.11)	0.90 (0.10)	0.82 (0.10)	1.01 (0.11)	1.06 (0.11)	1.18 (0.12)	1.15 (0.12)	0.95 (0.11)	0.93 (0.10)	4 (-23 to 42)
15-24	1.34 (0.10)	1.68 (0.11)	1.74 (0.11)	1.62 (0.11)	1.55 (0.11)	1.61 (0.11)	1.67 (0.11)	1.63 (0.11)	1.51 (0.11)	1.12 (0.09)	0.88 (0.08)	-34 (-48 to -17)
25-44	3.45 (0.12)	3.69 (0.12)	4.17 (0.13)	3.74 (0.12)	3.95 (0.13)	3.95 (0.12)	3.71 (0.12)	3.53 (0.12)	3.56 (0.12)	3.22 (0.11)	2.94 (0.11)	-15 (-23 to -6)
45-64	6.04 (0.19)	6.49 (0.19)	7.54 (0.21)	6.80 (0.19)	7.59 (0.20)	7.31 (0.19)	7.75 (0.19)	7.45 (0.18)	7.41 (0.18)	7.17 (0.18)	7.80 (0.18)	29 (20 to 39)
65-74	16.49 (0.63)	15.95 (0.61)	16.28 (0.62)	14.42 (0.58)	15.42 (0.60)	13.64 (0.53)	15.77 (0.58)	14.80 (0.56)	16.37 (0.58)	17.32 (0.60)	16.83 (0.55)	2 (-8 to 13)
75+	108.8 (2.33)	108.20 (2.30)	111.41 (2.30)	111.10 (2.25)	109.37 (2.20)	99.78 (1.97)	96.45 (1.81)	92.87 (1.73)	97.04 (1.73)	110.92 (1.84)	114.69 (1.76)	5 (0 to 11)
**Mechanism:**												
FSLS	1.60 (0.05)	1.77 (0.05)	2.02 (0.05)	2.06 (0.05)	2.19 (0.05)	2.08 (0.05)	2.16 (0.05)	2.09 (0.05)	2.37 (0.05)	2.84 (0.06)	3.05 (0.06)	91 (78 to 104)
FRF	0.23 (0.02)	0.17 (0.02)	0.26 (0.02)	0.26 (0.02)	0.25 (0.02)	0.21 (0.02)	0.19 (0.02)	0.19 (0.01)	0.20 (0.02)	0.26 (0.02)	0.22 (0.02)	-3 (-21 to 19)
OFSL	0.62 (0.03)	0.60 (0.03)	0.75 (0.03)	0.56 (0.03)	0.68 (0.03)	0.71 (0.03)	0.64 (0.03)	0.64 (0.03)	0.60 (0.03)	0.60 (0.03)	0.60 (0.03)	-3 (-14 to 10)
FSS	0.49 (0.03)	0.48 (0.03)	0.89 (0.03)	0.91 (0.03)	0.71 (0.03)	0.65 (0.03)	0.67 (0.03)	0.65 (0.03)	0.66 (0.03)	0.61 (0.03)	0.56 (0.03)	16 (1 to 32)
FBS	1.40 (0.04)	1.52 (0.04)	1.68 (0.05)	1.55 (0.04)	1.66 (0.05)	1.63 (0.04)	1.68 (0.05)	1.60 (0.04)	1.56 (0.04)	1.34 (0.04)	1.27 (0.04)	-9 (-17 to -1)
OFOL	1.57 (0.05)	1.55 (0.04)	1.59 (0.05)	1.47 (0.04)	1.60 (0.05)	1.55 (0.04)	1.46 (0.04)	1.46 (0.04)	1.44 (0.04)	1.39 (0.04)	1.42 (0.04)	-9 (-16 to -2)
UF	1.74 (0.05)	1.75 (0.05)	1.21 (0.04)	1.11 (0.04)	1.06 (0.04)	0.90 (0.03)	0.95 (0.03)	0.82 (0.03)	0.85 (0.03)	0.90 (0.03)	0.90 (0.03)	-48 (-53 to -44)

CI – confidence interval, FSLS – falls on same level from slipping, tripping and stumbling (W01), FRF – furniture related falls (W06-W08) OFSL – other falls on same level (W00, W02-W05, W09 and W18) FSS – falls on and from stairs and steps (W10), FBS – falls from, out of or through building or structure (W13) OFOL – other falls from one level to another (W11, W12 and W14-W17), UF – unspecified falls (W19).

*The overall and subgroup unintentional falls rates (excluding age groups) were age-adjusted using the population of China in 2010.

†% change in rate and its 95% CI that was calculated as “mortality rate ratio (MRR) – 1”.

### Mortality differences from location, sex and age group

Subgroup analysis by location (urban/rural), sex and age group displayed a trend for change that was highly similar to the overall age-standardized trend from 2006 to 2016, with the single exception of a notable decrease in fatal falls between 2012 and 2016 for the 15-24 years age group (**Figure 1**, panels A, B and C).

Across the study time period, males and rural residents consistently had higher unintentional falls mortality rates than females (male/female mortality rate ratio: 1.7-2.0) and urban residents (rural/urban mortality rate ratio: 1.3-1.4). The urban-rural mortality disparity varied somewhat across age groups (typically higher disparities appeared in the middle age groups) and changed modestly over the years of the study (Table S1 in **Online Supplementary Document**).

Male-female mortality differences were highest among the age groups 15-24 years (mortality rate ratio: 3.2-5.4), 25-44 years (mortality rate ratio: 4.9-6.2) and 45-64 years (mortality rate ratio: 3.6-5.0), and were lowest among age groups 0-4 years (mortality rate ratio: -1.1 to -2.6) and ≥75 years (mortality rate ratio: 0.9-1.0) (Table S2 in **Online Supplementary Document**).

For individuals aged 5 years and older, unintentional falls mortality increased quickly as age got older, and more than 44% of falls deaths occurred in the age group of 75 years and older from 2006 to 2016 (**Figure 1**, panel C; and **Table 1**). Children aged 0-4 years also had moderately high mortality (from 1.90-2.33 per 100 000 population). Unintentional fall mortality changes varied across the age groups during the study period. Significant mortality changes included a 29% increase in the age group 45-64 years, a 5% increase in the age group ≥75 years, and a decrease of over 15% in the combined age group of 15-44 years (**Table 1**).

### Unintentional falls mortality by mechanism

From 2006 to 2016, falls on the same level from slipping, tripping and stumbling (W01); falls from, out of or through building or structure (W13); and other falls from one level to another (W11, W12, W14-W17) were the most three common mechanisms of falls mortality, accounting respectively for 29%, 20% and 19% of total mortality. The age-standardized mortality rates for five mechanisms of falls showed roughly similar patterns of change over time from 2006 to 2016 as the overall age-standardized unintentional falls mortality (**Figure 1**, panel D). The remaining two mechanisms of falls, falls on the same level from slipping, tripping and stumbling (W01) and unspecified falls (W19), showed different patterns of change over time. Falls on the same level from slipping, tripping and stumbling (W01) rose from 1.60 to 3.05 per 100 000 population between 2006 and 2016, with an especially notable increase after 2013. Age-standardized mortality due to unspecified falls (W19) decreased from 1.74 to 0.90 per 100 000 (**Table 1**) between 2006 and 2016.

**Figure 2:**
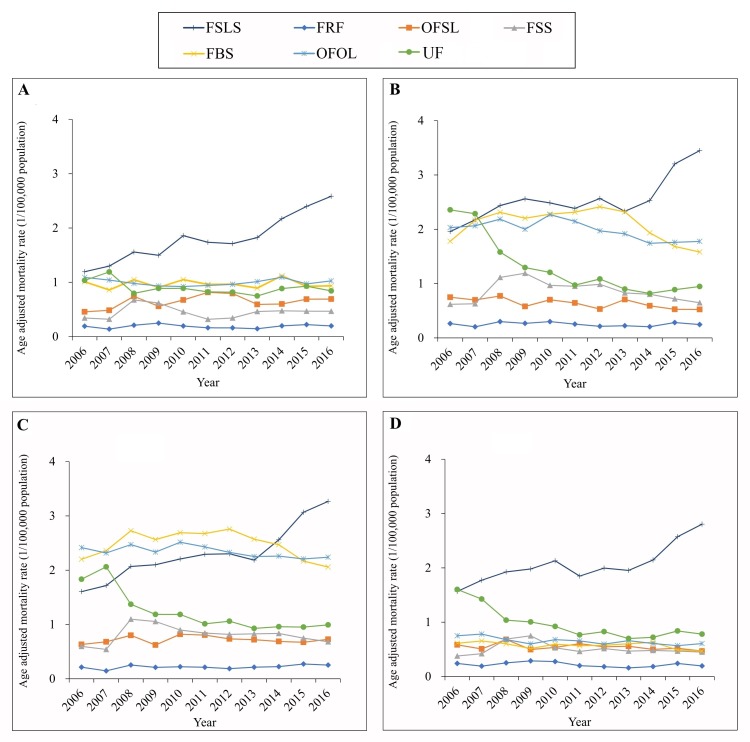
Mortality rates from unintentional falls by urban/rural, sex and mechanism in China, 2006-2016. Urban (panel **A**), rural (panel **B**), male (panel **C**), female (panel **D**). Mortality rates were age-standardized based on the population of China in 2010. FSLS – falls on same level from slipping, tripping and stumbling (W01), FRF – furniture related falls (W06-W08), OFSL – other falls on same level (W00, W02-W05, W09 and W18), FSS – falls on and from stairs and steps (W10), FBS – falls from, out of or through building or structure (W13), OFOL – other falls from one level to another (W11, W12 and W14-W17), UF – unspecified falls (W19).

Males and individuals from rural areas had much higher mortality rates from falls from, out of or through building or structure (W13) and from other falls from one level to another (W11, W12, W14-W17) compared to females and individuals from urban areas between 2006 and 2016 (**Figure 2**; and Tables S3 and S4 in **Online Supplementary Document**). Mortality rates from furniture related falls (W06-W08) were generally comparable between subsets of the population.

**Figure 3:**
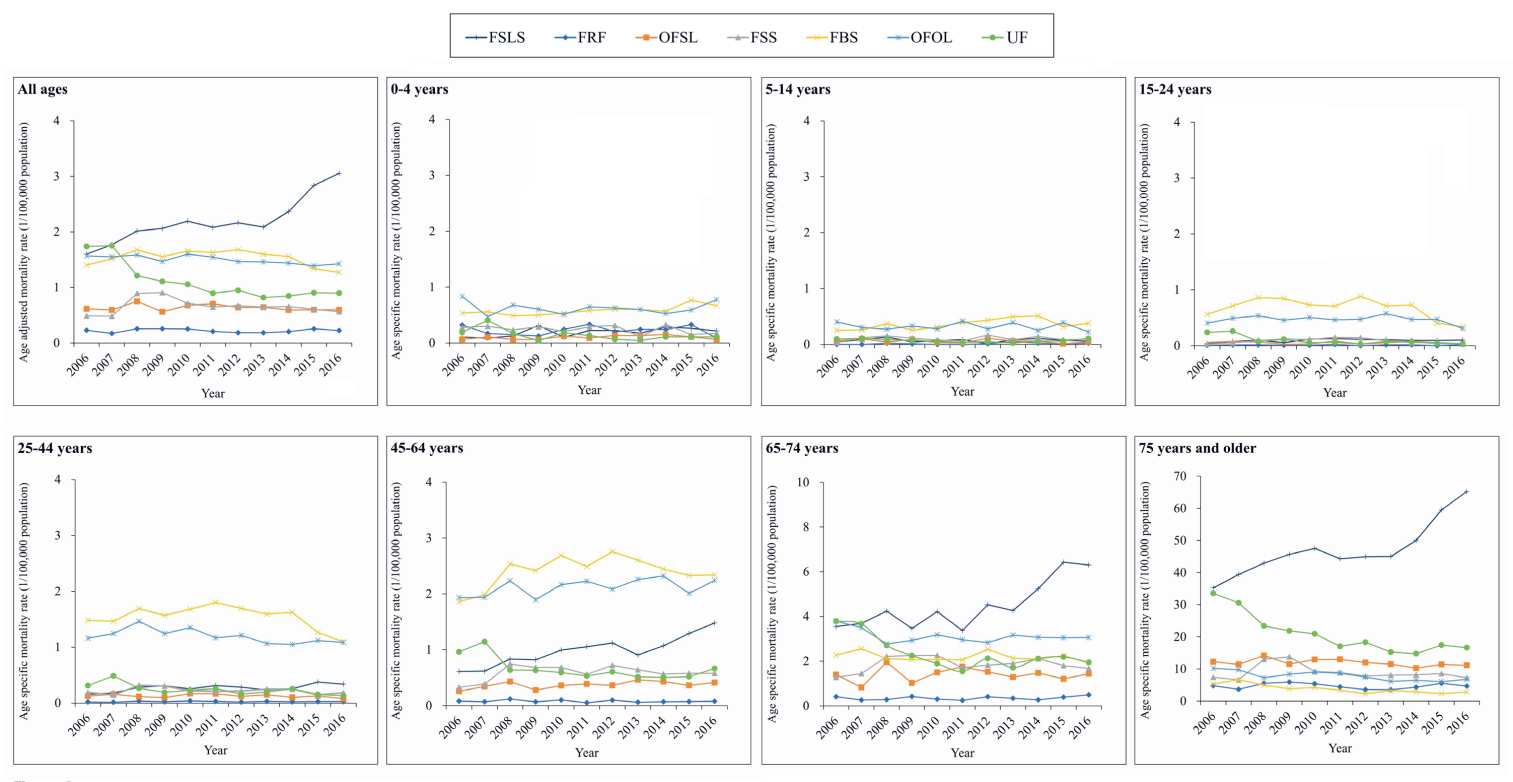
Mortality rates from unintentional falls by age group and mechanism in China, 2006-2016. FSLS – falls on same level from slipping, tripping and stumbling (W01), FRF – furniture related falls (W06-W08), OFSL – other falls on same level (W00, W02-W05, W09 and W18), FSS – falls on and from stairs and steps (W10), FBS – falls from, out of or through building or structure (W13), OFOL – other falls from one level to another (W11, W12 and W14-W17), UF – unspecified falls (W19).

**Figure 3** and Table S5 in **Online Supplementary Document** shows that the spectrum of unintentional falls mortality mechanisms changed greatly across age groups. For the five age groups under 65 years old, falls from, out of or through building or structure (W13) and other falls from one level to another (W11, W12, W14-W17) were the two leading mechanisms of unintentional falls mortality (Table S5 in **Online Supplementary Document**). For the two oldest age groups (65-74 years and ≥75 years), falls on the same level from slipping, tripping and stumbling (W01) was the leading mechanism of unintentional falls mortality, and mortality rates from these causes increased by 78% (from 3.55 to 6.30 per 100 000 population) and 85% (from 35.25 to 65.26 per 100 000 population) for the two age groups respectively between 2006 and 2016.

## DISCUSSION

This study presents three key findings: (a) age-adjusted unintentional falls mortality in China remained relatively high and increased slightly from 2006 to 2016; (b) males, rural residents and people aged ≥75 years consistently had a higher unintentional falls mortality rate than females, urban residents and other age groups; (c) in descending order, the three leading mechanisms of fatal unintentional falls were falls on the same level from slipping, tripping and stumbling; falls from, out of or through building or structure; and other falls from one level to another.

These data represent some of the most comprehensive data on the circumstances and trends for fatal unintentional falls across all of China. The crude mortality from unintentional falls was 9.55 per 100 000 population in 2016, significantly higher than that in rural Bangladesh (5.0 per 100 000 population in 2013) [19], but lower than data reported from the USA (10.61 per 100 000 population in 2016) [10] and India (14.5 per 100 000 population in 2005) [20]. Disparities across countries likely reflect the combined impact of differences in population ageing, geographically- and culturally-based risk factors, data reporting quality, and injury prevention efforts between nations. Recent studies indicate, for example, that mortality data reporting recently improved for unintentional falls among Americans ages 65 years and older [21-23].

One notable result from our study is that the overall unintentional falls mortality rate remained relatively stable and even increased slightly during the study period. This finding contrasts with recent improvements in other health outcome domains in China, such as infectious diseases and non-communicable chronic diseases [24], and may reflect comparative inattention to injury control efforts in China [25-27].

Our study replicates results from previous reports [18-20,28] that males, rural residents and older adults had higher falls mortality risk than females, urban residents and younger adults. Greater falls mortality risk in males and rural residents has been linked to higher likelihood of working in high-risk occupations, [29,30] which corresponds to the risky mechanisms for fatal falls that we identified – in particular to risk of falls from, out of or through buildings or structures. In addition, rural residents in China may face less safe working conditions and comparatively poor prehospital and hospital treatment services [31]. The increased risk of fatal falls among older adults is well documented in the literature and attributed to physical and cognitive limitations that are associated with aging. The increased risk of falls is also linked to having multiple non-communicable chronic diseases [32] and medication side-effects [33] among older adults.

One notable result we discovered was that for adults aged 25 years and older, a remarkable increase was observed in falls mortality from unintentional falls on the same level from slipping, tripping and stumbling (over 78%). This increase may be due to the absence of a single lead agency and a top-down professional team to take change of injury prevention in China, [25,26] as well as diminished agility among the growing population of older adults [30]. In addition, we discovered a significant increase in fall mortality from unintentional falls from, out of or through a building or structure among adults aged 45-64 years. This may be related to rapid economic growth over the past two decades in China, which involves substantial numbers of workers in the construction industry. Many older construction workers work in large numbers in the industry [34], and they lack appropriate fall protection devices and techniques [30], violate operational regulations, and lack necessary knowledge about occupational safety precautions [35,36].

Subgroup analyses showed different patterns for the mechanism of fatal unintentional falls between age groups 5-64 years and ≥65 years. For example, “falls out of or through building or structure” was the most common mechanism for Chinese people aged 5-64 years, while “falls on same level from slipping, tripping and stumbling” was most common for adults aged ≥65 years in China. This finding differs from reports from southern Sweden [37] and the USA [22], where the most common fatal falls are those coded as unspecified falls. The difference may result to some degree from differences in data reporting practice; we observed a 48% mortality decrease from falls with unspecified codes between 2006 and 2016, suggesting data reporting practice changes in China. The difference may also reflect discrepancies in risk exposure and falls prevention efforts between China and other countries.

Our findings have two implications. First, injury morbidity and mortality continue to be a major health challenge in China. These findings, in conjunction with previous reports concerning mortality from traumatic brain injury [16] and drowning [38], and overall injury morbidity [39], underscore the importance and urgency of implementing injury control measures in China. To realize the “Healthy China 2030” and Sustainable Development Goals (SDGs), Chinese society must address the major causes of fatal falls through empirically supported and theory-driven prevention practices. Many such programs exist and could be initiated in China; as an example, elderly falls prevention can be accomplished through community-implemented multifactorial interventions that incorporate education, risk assessment and suggestion, and exercise [40].

Second, our study offers a previously undocumented breakdown of the mechanisms and trends over time for unintentional falls mortality in China by sex, location and age group. The high-risk populations and major mechanisms for fall mortality identified in our study should be prioritized for fall prevention strategies in China. Central and local governments may consider establishing new laws or regulations if they do not exist, or revising existing ones, to enhance protection of vulnerable populations. As an example, stricter regulations and enforcement concerning safety protection equipment and safety inspections might protect workers from falling out of or through buildings or structures in the construction industry.

This study was primarily limited by the quality of the DSPs data. Although many efforts are made to verify data quality, and although the data set is widely used, the DSPs data are still potentially affected by underreporting and misclassification in reporting practice. Second, as is the case with almost all public health surveillance systems, the DSPs data do not collect detailed information on potential causal or mediating factors for falls, such as environmental risks, individual behavior, use of protective equipment, or relevant policy variables. In addition, we did not consider nonfatal falls data because nonfatal falls morbidity data are unavailable in the DSPs system.

## Additional Material

Online Supplementary Document
