# Supplementary material for: Unintentional falls mortality in China, 2006-2016
Source: J Glob Health. 2019 Mar 30;9(1):010603. doi: 10.7189/jogh.09.010603 (PMC6445498; doi:10.7189/jogh.09.010603)
Supplement: Online Supplementary Document [file jogh-09-010603-s001.pdf]

J Glob Health 2019; 9: 010603

Supplementary Table 1. Crude unintentional falls mortality per 100,000 population by age group and location in China, 2006-2016

Age group	Location	2006	2007	2008	2009	2010	2011	2012	2013	2014	2015	2016	%change in rate
**0-4 years**	Urban	1.63 (0.26)	1.32 (0.23)	0.98 (0.2)	0.76 (0.18)	1.34 (0.23)	1.07 (0.21)	0.87 (0.19)	1.18 (0.22)	1.93 (0.35)	1.65 (0.32)	1.94 (0.34)	19 (-25, 90)
	Rural	3.07 (0.37)	2.95 (0.36)	2.91 (0.36)	3.21 (0.37)	2.51 (0.33)	3.64 (0.41)	3.72 (0.43)	2.78 (0.37)	2.36 (0.28)	2.68 (0.3)	2.17 (0.27)	-29 (-50, -1)
	**Ratio**	**1.9**	**2.2**	**3.0**	**4.2**	**1.9**	**3.4**	**4.3**	**2.4**	**1.2**	**1.6**	**1.1**	
**5-14 years**	Urban	0.38 (0.09)	0.53 (0.1)	0.68 (0.12)	0.73 (0.12)	0.61 (0.11)	0.6 (0.12)	0.6 (0.12)	0.73 (0.13)	0.99 (0.18)	0.66 (0.14)	0.79 (0.16)	107 (15, 274)
	Rural	1.45 (0.18)	1.47 (0.18)	1.53 (0.19)	1.1 (0.16)	1.06 (0.16)	1.48 (0.2)	1.58 (0.2)	1.7 (0.21)	1.24 (0.15)	1.12 (0.15)	1.01 (0.14)	-30 (-51, 0)
	**Ratio**	**3.8**	**2.8**	**2.3**	**1.5**	**1.7**	**2.5**	**2.6**	**2.3**	**1.3**	**1.7**	**1.3**	
**15-24 years**	Urban	0.9 (0.11)	0.83 (0.11)	0.87 (0.11)	0.89 (0.11)	0.88 (0.11)	0.99 (0.12)	0.81 (0.11)	0.91 (0.12)	0.93 (0.12)	0.8 (0.11)	0.62 (0.1)	-30 (-53, 4)
	Rural	1.79 (0.16)	2.58 (0.2)	2.68 (0.2)	2.41 (0.2)	2.29 (0.19)	2.28 (0.19)	2.57 (0.2)	2.39 (0.19)	2.03 (0.17)	1.42 (0.15)	1.12 (0.13)	-37 (-53, -16)
	**Ratio**	**2.0**	**3.1**	**3.1**	**2.7**	**2.6**	**2.3**	**3.2**	**2.6**	**2.2**	**1.8**	**1.8**	
**25-44 years**	Urban	2.4 (0.14)	2.21 (0.13)	2.67 (0.14)	2.38 (0.14)	2.43 (0.14)	2.28 (0.13)	2.28 (0.13)	2.05 (0.12)	2.39 (0.14)	1.98 (0.13)	1.68 (0.11)	-30 (-41, -17)
	Rural	4.5 (0.19)	5.23 (0.2)	5.73 (0.21)	5.15 (0.2)	5.52 (0.21)	5.68 (0.21)	5.18 (0.2)	5.05 (0.2)	4.65 (0.19)	4.4 (0.18)	4.18 (0.18)	-7 (-17, 4)
	**Ratio**	**1.9**	**2.4**	**2.1**	**2.2**	**2.3**	**2.5**	**2.3**	**2.5**	**2.0**	**2.2**	**2.5**	
**45-64 years**	Urban	3.64 (0.21)	3.9 (0.21)	4.5 (0.22)	3.76 (0.2)	4.67 (0.22)	4.26 (0.2)	4.68 (0.21)	4.41 (0.19)	5.31 (0.22)	4.97 (0.22)	5.6 (0.23)	54 (34, 76)
	Rural	8.46 (0.32)	9.18 (0.33)	10.68 (0.35)	9.94 (0.33)	10.6 (0.33)	10.47 (0.32)	10.94 (0.32)	10.6 (0.31)	9.19 (0.27)	9.04 (0.27)	9.7 (0.28)	15 (5, 26)
	**Ratio**	**2.3**	**2.4**	**2.4**	**2.6**	**2.3**	**2.5**	**2.3**	**2.4**	**1.7**	**1.8**	**1.7**	
**65-74 years**	Urban	9.07 (0.67)	8.86 (0.65)	10.06 (0.69)	9.79 (0.68)	9.52 (0.67)	9.27 (0.63)	10.08 (0.67)	11.23 (0.7)	13.65 (0.81)	15.34 (0.85)	14.55 (0.79)	60 (34, 92)
	Rural	23.54 (1.05)	22.87 (1.03)	22.33 (1.01)	18.9 (0.93)	21.12 (0.98)	17.69 (0.84)	21.18 (0.94)	18.18 (0.86)	18.44 (0.82)	18.83 (0.82)	18.54 (0.77)	-21 (-30, -11)
	**Ratio**	**2.6**	**2.6**	**2.2**	**1.9**	**2.2**	**1.9**	**2.1**	**1.6**	**1.4**	**1.2**	**1.3**	
**75+ years**	Urban	84.84 (3.15)	85.88 (3.1)	94.94 (3.21)	93.08 (3.1)	97.77 (3.13)	94.02 (2.93)	90.68 (2.65)	89.33 (2.55)	98.99 (2.6)	106.58 (2.69)	110.44 (2.58)	30 (19, 42)
	Rural	126.81 (3.33)	125.59 (3.31)	124.27 (3.24)	125.26 (3.19)	118.49 (3.06)	104.05 (2.65)	100.96 (2.47)	95.66 (2.34)	95.44 (2.31)	114.45 (2.51)	118.13 (2.4)	-7 (-13, -1)
	**Ratio**	**1.5**	**1.5**	**1.3**	**1.4**	**1.2**	**1.1**	**1.1**	**1.1**	**1.0**	**1.1**	**1.1**	

Notes:

1. *: % change in rate and its 95% CI that was calculated as “mortality rate ratio (MRR) -1”;

2. Abbreviations: FSLS: Falls on same level from slipping, tripping and stumbling (W01); FRF: Furniture related falls (W06-W08); OFSL: other falls on same level (W00, W02-W05, W09 and W18); FSS: Falls on and from stairs and steps (W10); FBS: falls from, out of or through building or structure (W13); OFOL: other falls from one level to another (W11, W12 and W14-W17); UF: unspecified falls(W19).

Supplementary Table 2. Crude unintentional falls mortality per 100,000 population by age group and sex in China, 2006-2016

Age group	Sex	2006	2007	2008	2009	2010	2011	2012	2013	2014	2015	2016	%change in rate
**0-4 years**	Male	2.85 (0.35)	2.31 (0.31)	2.45 (0.32)	2.13 (0.29)	2.33 (0.3)	3.26 (0.37)	2.44 (0.32)	2.16 (0.3)	2.29 (0.3)	2.74 (0.33)	2.49 (0.31)	-12 (-38, 23)
	Female	1.80 (0.28)	1.87 (0.29)	1.32 (0.24)	1.74 (0.27)	1.45 (0.25)	1.26 (0.24)	1.86 (0.3)	1.61 (0.28)	2.12 (0.32)	1.83 (0.3)	1.61 (0.28)	-10 (-43, 41)
	**Ratio**	**1.6**	**1.2**	**1.9**	**1.2**	**1.6**	**2.6**	**1.3**	**1.4**	**1.1**	**1.5**	**1.6**	
**5-14 years**	Male	1.15 (0.15)	1.4 (0.17)	1.48 (0.17)	1.14 (0.15)	1.15 (0.16)	1.36 (0.18)	1.43 (0.18)	1.32 (0.17)	1.44 (0.18)	1.16 (0.16)	1.17 (0.16)	1 (-30, 47)
	Female	0.60 (0.12)	0.48 (0.11)	0.63 (0.12)	0.63 (0.12)	0.45 (0.1)	0.62 (0.13)	0.62 (0.13)	1.01 (0.16)	0.81 (0.14)	0.71 (0.13)	0.65 (0.13)	9 (-37, 86)
	**Ratio**	**1.9**	**2.9**	**2.4**	**1.8**	**2.6**	**2.2**	**2.3**	**1.3**	**1.8**	**1.6**	**1.8**	
**15-24 years**	Male	2.13 (0.18)	2.72 (0.2)	2.84 (0.2)	2.67 (0.2)	2.46 (0.19)	2.56 (0.19)	2.68 (0.2)	2.45 (0.19)	2.39 (0.19)	1.73 (0.16)	1.35 (0.14)	-37 (-51, -18)
	Female	0.52 (0.09)	0.57 (0.09)	0.56 (0.09)	0.49 (0.09)	0.57 (0.1)	0.58 (0.09)	0.63 (0.1)	0.77 (0.11)	0.59 (0.1)	0.48 (0.09)	0.38 (0.08)	-27 (-57, 24)
	**Ratio**	**4.1**	**4.8**	**5.0**	**5.4**	**4.3**	**4.5**	**4.3**	**3.2**	**4.0**	**3.6**	**3.6**	
**25-44 years**	Male	5.85 (0.21)	6.09 (0.22)	7.1 (0.23)	6.36 (0.22)	6.58 (0.23)	6.5 (0.22)	6.31 (0.22)	5.88 (0.21)	5.87 (0.21)	5.35 (0.2)	4.93 (0.19)	-16 (-24, -6)
	Female	1.06 (0.09)	1.22 (0.1)	1.16 (0.1)	1.04 (0.09)	1.23 (0.1)	1.32 (0.1)	1.02 (0.09)	1.1 (0.09)	1.19 (0.1)	1.05 (0.09)	0.89 (0.08)	-16 (-34, 8)
	**Ratio**	**5.5**	**5.0**	**6.1**	**6.1**	**5.3**	**4.9**	**6.2**	**5.3**	**4.9**	**5.1**	**5.5**	
**45-64 years**	Male	9.39 (0.33)	10.05 (0.34)	11.87 (0.36)	10.97 (0.34)	12.29 (0.35)	12.01 (0.33)	12.33 (0.34)	12.09 (0.32)	12.13 (0.32)	11.71 (0.32)	12.52 (0.33)	33 (22, 45)
	Female	2.65 (0.18)	2.79 (0.18)	3.04 (0.19)	2.50 (0.16)	2.74 (0.17)	2.43 (0.15)	2.99 (0.17)	2.65 (0.15)	2.54 (0.15)	2.51 (0.15)	2.91 (0.16)	10 (-7, 30)
	**Ratio**	**3.6**	**3.6**	**3.9**	**4.4**	**4.5**	**5.0**	**4.1**	**4.6**	**4.8**	**4.7**	**4.3**	
**65-74 years**	Male	21.41 (1.03)	20.02 (0.98)	21.58 (1.01)	20.86 (0.99)	20.74 (0.99)	18.45 (0.89)	21.56 (0.97)	20.29 (0.94)	21.5 (0.96)	24.6 (1.02)	23.56 (0.94)	10 (-3, 24)
	Female	11.82 (0.75)	11.97 (0.75)	11.12 (0.71)	8.19 (0.61)	10.29 (0.68)	9.16 (0.6)	10.26 (0.66)	9.58 (0.63)	11.49 (0.68)	10.5 (0.65)	10.54 (0.61)	-11 (-25, 5)
	**Ratio**	**1.8**	**1.7**	**1.9**	**2.6**	**2.0**	**2.0**	**2.1**	**2.1**	**1.9**	**2.3**	**2.2**	
**75+ years**	Male	102.34 (3.46)	104.04 (3.42)	106.11 (3.39)	102.26 (3.25)	105.19 (3.25)	101.05 (3.04)	93.69 (2.69)	89.18 (2.55)	95.57 (2.58)	104.83 (2.69)	111.03 (2.61)	8 (0, 18)
	Female	113.65 (3.16)	111.44 (3.12)	115.56 (3.13)	118.04 (3.09)	112.66 (2.98)	98.83 (2.59)	98.6 (2.44)	95.77 (2.35)	98.22 (2.33)	115.71 (2.51)	117.56 (2.38)	3 (-3, 11)
	**Ratio**	**0.9**	**0.9**	**0.9**	**0.9**	**0.9**	**1.0**	**1.0**	**0.9**	**1.0**	**0.9**	**0.9**	

Notes:

1. *: % change in rate and its 95% CI that was calculated as “mortality rate ratio (MRR) -1”;

2. Abbreviations: FSLS: Falls on same level from slipping, tripping and stumbling (W01); FRF: Furniture related falls (W06-W08); OFSL: other falls on same level (W00, W02-W05, W09 and W18); FSS: Falls on and from stairs and steps (W10); FBS: falls from, out of or through building or structure (W13); OFOL: other falls from one level to another (W11, W12 and W14-W17); UF: unspecified falls(W19).

Supplementary Table 3. Mortality rate from unintentional falls per 100,000 population (standard error) by mechanism and location in China, 2006-2016

Mechanism	Location	2006	2007	2008	2009	2010	2011	2012	2013	2014	2015	2016	%change in rate (95% CI)
**FSLS**	Urban	1.19 (0.06)	1.3 (0.07)	1.56 (0.07)	1.50 (0.07)	1.86 (0.08)	1.74 (0.07)	1.71 (0.07)	1.82 (0.07)	2.17 (0.08)	2.40 (0.08)	2.58 (0.08)	116 (91, 145)
	Rural	1.96 (0.06)	2.17 (0.07)	2.44 (0.07)	2.56 (0.07)	2.49 (0.07)	2.38 (0.07)	2.57 (0.07)	2.33 (0.07)	2.53 (0.07)	3.20 (0.08)	3.45 (0.09)	76 (62, 91)
	**Ratio**	**1.6**	**1.7**	**1.6**	**1.7**	**1.3**	**1.4**	**1.5**	**1.3**	**1.2**	**1.3**	**1.3**	
**FRF**	Urban	0.19 (0.03)	0.14 (0.02)	0.21 (0.03)	0.25 (0.03)	0.2 (0.03)	0.16 (0.02)	0.16 (0.02)	0.15 (0.02)	0.20 (0.02)	0.22 (0.02)	0.20 (0.02)	2 (-28, 44)
	Rural	0.26 (0.02)	0.2 (0.02)	0.3 (0.02)	0.27 (0.02)	0.3 (0.02)	0.25 (0.02)	0.21 (0.02)	0.22 (0.02)	0.21 (0.02)	0.28 (0.03)	0.25 (0.02)	-7 (-28, 20)
	**Ratio**	**1.4**	**1.4**	**1.4**	**1.1**	**1.5**	**1.6**	**1.3**	**1.5**	**1.1**	**1.3**	**1.3**	
**OFSL**	Urban	0.46 (0.04)	0.48 (0.04)	0.74 (0.05)	0.56 (0.04)	0.67 (0.05)	0.82 (0.05)	0.79 (0.05)	0.59 (0.04)	0.6 (0.04)	0.69 (0.04)	0.69 (0.04)	52 (23, 87)
	Rural	0.75 (0.04)	0.7 (0.04)	0.77 (0.04)	0.58 (0.03)	0.7 (0.04)	0.64 (0.04)	0.53 (0.03)	0.71 (0.04)	0.59 (0.04)	0.53 (0.03)	0.52 (0.03)	-30 (-41, -17)
	**Ratio**	**1.6**	**1.5**	**1.0**	**1.0**	**1.0**	**0.8**	**0.7**	**1.2**	**1.0**	**0.8**	**0.8**	
**FSS**	Urban	0.34 (0.03)	0.32 (0.03)	0.68 (0.05)	0.62 (0.05)	0.46 (0.04)	0.32 (0.03)	0.34 (0.03)	0.46 (0.04)	0.48 (0.04)	0.47 (0.04)	0.47 (0.03)	36 (6, 74)
	Rural	0.62 (0.04)	0.63 (0.04)	1.11 (0.05)	1.19 (0.05)	0.97 (0.04)	0.95 (0.05)	0.99 (0.05)	0.83 (0.04)	0.8 (0.04)	0.72 (0.04)	0.65 (0.04)	5 (-11, 23)
	**Ratio**	**1.8**	**2.0**	**1.6**	**1.9**	**2.1**	**3.0**	**2.9**	**1.8**	**1.7**	**1.5**	**1.4**	
**FBS**	Urban	1.01 (0.06)	0.86 (0.05)	1.05 (0.06)	0.91 (0.06)	1.05 (0.06)	0.96 (0.05)	0.97 (0.05)	0.89 (0.05)	1.12 (0.05)	0.93 (0.05)	0.94 (0.05)	-7 (-21, 8)
	Rural	1.78 (0.06)	2.17 (0.07)	2.31 (0.07)	2.20 (0.07)	2.28 (0.07)	2.32 (0.07)	2.41 (0.07)	2.32 (0.07)	1.93 (0.07)	1.68 (0.06)	1.58 (0.06)	-11 (-20, -2)
	**Ratio**	**1.8**	**2.5**	**2.2**	**2.4**	**2.2**	**2.4**	**2.5**	**2.6**	**1.7**	**1.8**	**1.7**	
**OFOL**	Urban	1.09 (0.06)	1.04 (0.06)	0.98 (0.06)	0.93 (0.06)	0.92 (0.06)	0.94 (0.05)	0.96 (0.05)	1.01 (0.05)	1.09 (0.05)	0.97 (0.05)	1.03 (0.05)	-6 (-19, 9)
	Rural	2.03 (0.07)	2.07 (0.07)	2.19 (0.07)	2.00 (0.06)	2.27 (0.07)	2.15 (0.07)	1.97 (0.07)	1.92 (0.06)	1.74 (0.06)	1.76 (0.06)	1.78 (0.06)	-12 (-20, -4)
	**Ratio**	**1.9**	**2.0**	**2.2**	**2.2**	**2.5**	**2.3**	**2.1**	**1.9**	**1.6**	**1.8**	**1.7**	
**UF**	Urban	1.04 (0.06)	1.19 (0.06)	0.80 (0.05)	0.89 (0.05)	0.89 (0.06)	0.82 (0.05)	0.82 (0.05)	0.75 (0.05)	0.88 (0.05)	0.93 (0.05)	0.84 (0.05)	-19 (-30, -5)
	Rural	2.36 (0.07)	2.29 (0.07)	1.58 (0.06)	1.30 (0.05)	1.21 (0.05)	0.97 (0.05)	1.08 (0.05)	0.90 (0.04)	0.82 (0.04)	0.89 (0.04)	0.95 (0.05)	-60 (-64, -55)
	**Ratio**	**2.3**	**1.9**	**2.0**	**1.5**	**1.4**	**1.2**	**1.3**	**1.2**	**0.9**	**1.0**	**1.1**	

Notes:

1. % change in rate and its 95% CI that was calculated as “mortality rate ratio (MRR) -1”;

2. Abbreviations: FSLS: Falls on same level from slipping, tripping and stumbling (W01); FRF: Furniture related falls (W06-W08); OFSL: other falls on same level (W00, W02-W05, W09 and W18); FSS: Falls on and from stairs and steps (W10); FBS: falls from, out of or through building or structure (W13); OFOL: other falls from one level to another (W11, W12 and W14-W17); UF: unspecified falls(W19).

Supplementary Table 4. Mortality rate from unintentional falls per 100,000 population (standard error) by mechanism and sex in China, 2006-2016

Mechanism	Location	2006	2007	2008	2009	2010	2011	2012	2013	2014	2015	2016	%change in rate (95% CI)
**FSLS**	Male	1.61 (0.06)	1.72 (0.07)	2.07 (0.07)	2.1 (0.07)	2.21 (0.07)	2.29 (0.07)	2.30 (0.07)	2.19 (0.07)	2.56 (0.08)	3.07 (0.09)	3.27 (0.09)	103 (85, 124)
	Female	1.57 (0.06)	1.77 (0.07)	1.93 (0.07)	1.98 (0.07)	2.13 (0.07)	1.85 (0.07)	2.00 (0.07)	1.95 (0.07)	2.15 (0.07)	2.57 (0.08)	2.81 (0.08)	79 (62, 98)
	**Ratio**	**1.0**	**1.0**	**1.1**	**1.1**	**1.0**	**1.2**	**1.2**	**1.1**	**1.2**	**1.2**	**1.2**	
**FRF**	Male	0.21 (0.02)	0.15 (0.02)	0.25 (0.03)	0.21 (0.02)	0.22 (0.02)	0.21 (0.02)	0.19 (0.02)	0.21 (0.02)	0.22 (0.02)	0.27 (0.03)	0.25 (0.02)	18 (-11, 58)
	Female	0.24 (0.03)	0.19 (0.02)	0.25 (0.03)	0.29 (0.03)	0.28 (0.03)	0.2 (0.02)	0.18 (0.02)	0.16 (0.02)	0.18 (0.02)	0.24 (0.02)	0.19 (0.02)	-19 (-40, 10)
	**Ratio**	**0.9**	**0.8**	**1.0**	**0.7**	**0.8**	**1.1**	**1.1**	**1.3**	**1.2**	**1.1**	**1.3**	
**OFSL**	Male	0.63 (0.04)	0.68 (0.04)	0.80 (0.05)	0.62 (0.04)	0.82 (0.05)	0.81 (0.04)	0.74 (0.04)	0.72 (0.04)	0.69 (0.04)	0.67 (0.04)	0.73 (0.04)	15 (-3, 36)
	Female	0.58 (0.04)	0.51 (0.04)	0.68 (0.04)	0.50 (0.04)	0.54 (0.04)	0.60 (0.04)	0.55 (0.04)	0.56 (0.04)	0.50 (0.04)	0.52 (0.04)	0.47 (0.03)	-20 (-34, -3)
	**Ratio**	**1.1**	**1.3**	**1.2**	**1.2**	**1.5**	**1.4**	**1.3**	**1.3**	**1.4**	**1.3**	**1.6**	
**FSS**	Male	0.60 (0.04)	0.54 (0.04)	1.10 (0.05)	1.06 (0.05)	0.90 (0.05)	0.84 (0.05)	0.82 (0.04)	0.83 (0.04)	0.84 (0.04)	0.75 (0.04)	0.68 (0.04)	14 (-4, 36)
	Female	0.38 (0.03)	0.42 (0.03)	0.69 (0.04)	0.75 (0.04)	0.53 (0.04)	0.46 (0.03)	0.51 (0.04)	0.47 (0.03)	0.48 (0.03)	0.47 (0.03)	0.45 (0.03)	19 (-4, 49)
	**Ratio**	**1.6**	**1.3**	**1.6**	**1.4**	**1.7**	**1.8**	**1.6**	**1.8**	**1.8**	**1.6**	**1.5**	
**FBS**	Male	2.20 (0.08)	2.36 (0.08)	2.73 (0.08)	2.56 (0.08)	2.69 (0.08)	2.67 (0.08)	2.76 (0.08)	2.57 (0.08)	2.47 (0.08)	2.17 (0.07)	2.06 (0.07)	-7 (-15, 3)
	Female	0.61 (0.04)	0.65 (0.04)	0.60 (0.04)	0.51 (0.04)	0.59 (0.04)	0.57 (0.04)	0.57 (0.04)	0.60 (0.04)	0.63 (0.04)	0.49 (0.03)	0.47 (0.03)	-24 (-37, -7)
	**Ratio**	**3.6**	**3.6**	**4.6**	**5.0**	**4.6**	**4.7**	**4.8**	**4.3**	**3.9**	**4.4**	**4.4**	
**OFOL**	Male	2.42 (0.08)	2.32 (0.08)	2.47 (0.08)	2.33 (0.08)	2.51 (0.08)	2.43 (0.08)	2.33 (0.07)	2.25 (0.07)	2.26 (0.07)	2.21 (0.07)	2.24 (0.07)	-7 (-15, 1)
	Female	0.75 (0.04)	0.78 (0.05)	0.68 (0.04)	0.60 (0.04)	0.68 (0.04)	0.66 (0.04)	0.60 (0.04)	0.66 (0.04)	0.61 (0.04)	0.57 (0.04)	0.61 (0.04)	-19 (-32, -4)
	**Ratio**	**3.2**	**3.0**	**3.6**	**3.9**	**3.7**	**3.7**	**3.9**	**3.4**	**3.7**	**3.9**	**3.7**	
**UF**	Male	1.83 (0.07)	2.06 (0.07)	1.37 (0.06)	1.19 (0.05)	1.18 (0.05)	1.01 (0.05)	1.06 (0.05)	0.93 (0.05)	0.96 (0.05)	0.95 (0.05)	0.99 (0.05)	-46 (-52, -39)
	Female	1.60 (0.07)	1.43 (0.06)	1.04 (0.05)	1.00 (0.05)	0.92 (0.05)	0.77 (0.04)	0.83 (0.05)	0.70 (0.04)	0.72 (0.04)	0.84 (0.05)	0.78 (0.04)	-51 (-57, -44)
	**Ratio**	**1.1**	**1.4**	**1.3**	**1.2**	**1.3**	**1.3**	**1.3**	**1.3**	**1.3**	**1.1**	**1.3**	

Notes:

1. % change in rate and its 95% CI that was calculated as "mortality rate ratio (MRR) -1";

2. Abbreviations: FSLS: Falls on same level from slipping, tripping and stumbling (W01); FRF: Furniture related falls (W06-W08); OFSL: other falls on same level (W00, W02-W05, W09 and W18); FSS: Falls on and from stairs and steps (W10); FBS: falls from, out of or through building or structure (W13); OFOL: other falls from one level to another (W11, W12 and W14-W17); UF: unspecified falls(W19).

Supplementary Table 5. Unintentional falls mortality per 100,000 population (standard error) by age group and mechanism in China, 2006-2016

Age group	Mechanism	2006	2007	2008	2009	2010	2011	2012	2013	2014	2015	2016	%change in rate (95% CI)
0-4 years	FSLS	0.11 (0.05)	0.09 (0.04)	0.13 (0.05)	0.31 (0.08)	0.10 (0.05)	0.22 (0.07)	0.22 (0.07)	0.18 (0.06)	0.28 (0.08)	0.26 (0.08)	0.22 (0.07)	101 (-31, 489)
	FRF	0.32 (0.08)	0.17 (0.06)	0.15 (0.06)	0.13 (0.05)	0.25 (0.07)	0.34 (0.09)	0.20 (0.07)	0.24 (0.07)	0.24 (0.07)	0.33 (0.08)	0.09 (0.04)	-73 (-91, -19)
	OFSL	0.06 (0.04)	0.11 (0.05)	0.06 (0.04)	0.06 (0.04)	0.12 (0.05)	0.09 (0.04)	0.13 (0.05)	0.13 (0.05)	0.15 (0.06)	0.11 (0.05)	0.06 (0.04)	1 (-80, 399)
	FSS	0.28 (0.08)	0.3 (0.08)	0.23 (0.07)	0.29 (0.08)	0.20 (0.06)	0.29 (0.08)	0.31 (0.08)	0.11 (0.05)	0.33 (0.08)	0.15 (0.06)	0.17 (0.06)	-38 (-74, 49)
	FBS	0.54 (0.11)	0.56 (0.11)	0.49 (0.10)	0.50 (0.10)	0.53 (0.10)	0.58 (0.11)	0.61 (0.12)	0.6 (0.12)	0.57 (0.11)	0.77 (0.13)	0.67 (0.12)	25 (-26, 111)
	OFOL	0.84 (0.13)	0.47 (0.1)	0.68 (0.12)	0.61 (0.11)	0.51 (0.10)	0.65 (0.12)	0.63 (0.12)	0.6 (0.12)	0.53 (0.11)	0.59 (0.11)	0.78 (0.13)	-7 (-41, 46)
	UF	0.19 (0.06)	0.41 (0.09)	0.17 (0.06)	0.04 (0.03)	0.18 (0.06)	0.13 (0.05)	0.07 (0.04)	0.04 (0.03)	0.11 (0.05)	0.11 (0.05)	0.11 (0.05)	-44 (-81, 67)
5-14 years	FSLS	0.04 (0.02)	0.09 (0.03)	0.13 (0.04)	0.04 (0.02)	0.07 (0.03)	0.09 (0.03)	0.01 (0.01)	0.08 (0.03)	0.11 (0.04)	0.07 (0.03)	0.06 (0.03)	39 (-63, 416)
	FRF	-	-	0.02 (0.02)	0.01 (0.01)	-	-	0.01 (0.01)	0.05 (0.02)	0.02 (0.02)	0.01 (0.01)	0.02 (0.02)	-
	OFSL	0.06 (0.03)	0.1 (0.03)	0.04 (0.02)	0.09 (0.03)	0.03 (0.02)	0.02 (0.02)	0.11 (0.04)	0.06 (0.03)	0.06 (0.03)	0.01 (0.01)	0.05 (0.02)	-26 (-79, 162)
	FSS	0.03 (0.02)	0.12 (0.04)	0.15 (0.04)	0.11 (0.03)	0.08 (0.03)	0.06 (0.03)	0.17 (0.04)	0.08 (0.03)	0.14 (0.04)	0.08 (0.03)	0.09 (0.03)	195 (-22, 1014)
	FBS	0.25 (0.05)	0.26 (0.05)	0.37 (0.06)	0.25 (0.05)	0.31 (0.06)	0.39 (0.07)	0.43 (0.07)	0.5 (0.08)	0.51 (0.08)	0.32 (0.06)	0.38 (0.07)	48 (-13, 151)
	OFOL	0.40 (0.07)	0.30 (0.06)	0.27 (0.05)	0.33 (0.06)	0.28 (0.06)	0.42 (0.07)	0.28 (0.06)	0.39 (0.07)	0.25 (0.05)	0.39 (0.07)	0.22 (0.05)	-45 (-68, -4)
	UF	0.10 (0.03)	0.11 (0.03)	0.09 (0.03)	0.07 (0.03)	0.06 (0.02)	0.04 (0.02)	0.05 (0.02)	0.02 (0.02)	0.06 (0.03)	0.06 (0.03)	0.11 (0.04)	11 (-56, 179)
15-24 years	FSLS	0.04 (0.02)	0.07 (0.02)	0.10 (0.03)	0.05 (0.02)	0.12 (0.03)	0.13 (0.03)	0.11 (0.03)	0.11 (0.03)	0.09 (0.03)	0.09 (0.03)	0.10 (0.03)	168 (-5, 650)
	FRF	0.01 (0.01)	0.01 (0.01)	0.02 (0.01)	0.01 (0.01)	0.01 (0.01)	0.02 (0.01)	-	0.02 (0.01)	0.02 (0.01)	-	0.02 (0.01)	209 (-68, 2867)
	OFSL	0.06 (0.02)	0.07 (0.02)	0.07 (0.02)	0.02 (0.01)	0.03 (0.02)	0.08 (0.02)	0.03 (0.01)	0.05 (0.02)	0.08 (0.02)	0.06 (0.02)	0.04 (0.02)	-36 (-79, 97)
	FSS	0.03 (0.01)	0.05 (0.02)	0.09 (0.03)	0.12 (0.03)	0.11 (0.03)	0.15 (0.03)	0.14 (0.03)	0.09 (0.03)	0.05 (0.02)	0.06 (0.02)	0.03 (0.02)	3 (-74, 311)
	FBS	0.56 (0.06)	0.71 (0.07)	0.86 (0.08)	0.84 (0.08)	0.73 (0.08)	0.71 (0.07)	0.88 (0.08)	0.71 (0.07)	0.73 (0.08)	0.40 (0.06)	0.34 (0.05)	-40 (-58, -12)
	OFOL	0.40 (0.05)	0.49 (0.06)	0.54 (0.06)	0.46 (0.06)	0.5 (0.06)	0.46 (0.06)	0.47 (0.06)	0.57 (0.07)	0.47 (0.06)	0.47 (0.06)	0.31 (0.05)	-24 (-49, 15)
	UF	0.24 (0.04)	0.26 (0.04)	0.08 (0.02)	0.11 (0.03)	0.05 (0.02)	0.06 (0.02)	0.03 (0.01)	0.08 (0.02)	0.07 (0.02)	0.04 (0.02)	0.04 (0.02)	-84 (-94, -59)
25-44 years	FSLS	0.16 (0.02)	0.18 (0.03)	0.28 (0.03)	0.31 (0.03)	0.25 (0.03)	0.32 (0.03)	0.28 (0.03)	0.23 (0.03)	0.26 (0.03)	0.38 (0.04)	0.34 (0.04)	116 (48, 214)
	FRF	0.02 (0.01)	0.02 (0.01)	0.03 (0.01)	0.02 (0.01)	0.04 (0.01)	0.03 (0.01)	0.02 (0.01)	0.03 (0.01)	0.02 (0.01)	0.03 (0.01)	0.03 (0.01)	39 (-56, 338)
	OFSL	0.13 (0.02)	0.16 (0.02)	0.12 (0.02)	0.10 (0.02)	0.17 (0.03)	0.17 (0.02)	0.12 (0.02)	0.15 (0.02)	0.10 (0.02)	0.13 (0.02)	0.08 (0.02)	-38 (-65, 8)
	FSS	0.19 (0.03)	0.14 (0.02)	0.32 (0.04)	0.31 (0.03)	0.22 (0.03)	0.21 (0.03)	0.22 (0.03)	0.25 (0.03)	0.25 (0.03)	0.15 (0.02)	0.18 (0.03)	-5 (-36, 42)
	FBS	1.48 (0.08)	1.46 (0.08)	1.69 (0.08)	1.57 (0.08)	1.68 (0.08)	1.80 (0.08)	1.69 (0.08)	1.59 (0.08)	1.62 (0.08)	1.26 (0.07)	1.09 (0.07)	-26 (-37, -14)
	OFOL	1.16 (0.07)	1.24 (0.07)	1.46 (0.08)	1.24 (0.07)	1.35 (0.07)	1.17 (0.07)	1.21 (0.07)	1.07 (0.06)	1.05 (0.06)	1.12 (0.07)	1.09 (0.07)	-6 (-21, 10)
	UF	0.31 (0.04)	0.49 (0.04)	0.27 (0.03)	0.19 (0.03)	0.23 (0.03)	0.26 (0.03)	0.16 (0.02)	0.20 (0.03)	0.25 (0.03)	0.15 (0.02)	0.13 (0.02)	-58 (-72, -37)
45-64 years	FSLS	0.61 (0.06)	0.62 (0.06)	0.83 (0.07)	0.82 (0.07)	0.99 (0.07)	1.05 (0.07)	1.12 (0.07)	0.91 (0.06)	1.07 (0.07)	1.29 (0.07)	1.48 (0.08)	142 (95, 202)
	FRF	0.08 (0.02)	0.07 (0.02)	0.12 (0.03)	0.07 (0.02)	0.10 (0.02)	0.05 (0.02)	0.10 (0.02)	0.06 (0.02)	0.07 (0.02)	0.07 (0.02)	0.08 (0.02)	-5 (-53, 92)

	OFSL	0.26 (0.04)	0.34 (0.04)	0.43 (0.05)	0.28 (0.04)	0.36 (0.04)	0.39 (0.04)	0.36 (0.04)	0.46 (0.04)	0.43 (0.04)	0.36 (0.04)	0.41 (0.04)	60 (12, 129)
	FSS	0.33 (0.04)	0.39 (0.05)	0.74 (0.06)	0.68 (0.06)	0.68 (0.06)	0.56 (0.05)	0.72 (0.06)	0.64 (0.05)	0.57 (0.05)	0.58 (0.05)	0.58 (0.05)	76 (29, 141)
	FBS	1.86 (0.10)	1.99 (0.11)	2.54 (0.12)	2.42 (0.11)	2.69 (0.12)	2.49 (0.11)	2.76 (0.11)	2.60 (0.11)	2.45 (0.10)	2.33 (0.10)	2.34 (0.10)	26 (9, 44)
	OFOL	1.93 (0.11)	1.94 (0.11)	2.23 (0.11)	1.90 (0.10)	2.17 (0.10)	2.23 (0.10)	2.09 (0.1)	2.26 (0.10)	2.32 (0.10)	2.01 (0.09)	2.24 (0.10)	16 (1, 33)
	UF	0.96 (0.08)	1.14 (0.08)	0.64 (0.06)	0.63 (0.06)	0.59 (0.05)	0.53 (0.05)	0.61 (0.05)	0.52 (0.05)	0.51 (0.05)	0.52 (0.05)	0.67 (0.05)	-31 (-45, -14)
65-74 years	FSLS	3.55 (0.29)	3.69 (0.30)	4.24 (0.31)	3.47 (0.28)	4.22 (0.31)	3.37 (0.26)	4.53 (0.31)	4.26 (0.30)	5.24 (0.33)	6.43 (0.36)	6.30 (0.34)	78 (46, 115)
	FRF	0.41 (0.10)	0.26 (0.08)	0.28 (0.08)	0.42 (0.10)	0.30 (0.08)	0.25 (0.07)	0.41 (0.09)	0.34 (0.08)	0.27 (0.07)	0.39 (0.09)	0.49 (0.09)	20 (-35, 120)
	OFSL	1.40 (0.18)	0.83 (0.14)	1.96 (0.21)	1.02 (0.15)	1.50 (0.19)	1.75 (0.19)	1.52 (0.18)	1.29 (0.16)	1.47 (0.17)	1.21 (0.16)	1.44 (0.16)	3 (-27, 44)
	FSS	1.28 (0.18)	1.44 (0.18)	2.21 (0.23)	2.26 (0.23)	2.26 (0.23)	1.71 (0.19)	1.82 (0.20)	1.90 (0.20)	2.11 (0.21)	1.80 (0.19)	1.68 (0.17)	31 (-7, 84)
	FBS	2.27 (0.23)	2.56 (0.25)	2.12 (0.22)	2.07 (0.22)	2.07 (0.22)	2.06 (0.21)	2.53 (0.23)	2.13 (0.21)	2.09 (0.21)	2.25 (0.21)	1.91 (0.19)	-16 (-36, 11)
	OFOL	3.79 (0.30)	3.50 (0.29)	2.77 (0.25)	2.93 (0.26)	3.18 (0.27)	2.96 (0.25)	2.83 (0.25)	3.17 (0.26)	3.07 (0.25)	3.05 (0.25)	3.06 (0.24)	-19 (-35, 0)
	UF	3.79 (0.30)	3.67 (0.29)	2.70 (0.25)	2.26 (0.23)	1.89 (0.21)	1.54 (0.18)	2.12 (0.21)	1.71 (0.19)	2.11 (0.21)	2.19 (0.21)	1.95 (0.19)	-49 (-60, -34)
⩾75 years	FSLS	35.25 (1.33)	39.42 (1.39)	42.95 (1.43)	45.67 (1.44)	47.54 (1.45)	44.31 (1.31)	44.93 (1.23)	45.01 (1.20)	50.04 (1.24)	59.52 (1.35)	65.26 (1.32)	85 (70, 101)
	FRF	4.81 (0.49)	3.68 (0.43)	5.56 (0.51)	5.95 (0.52)	5.43 (0.49)	4.43 (0.42)	3.60 (0.35)	3.56 (0.34)	4.34 (0.37)	5.57 (0.41)	4.68 (0.35)	-3 (-24, 25)
	OFSL	12.27 (0.78)	11.44 (0.75)	14.21 (0.82)	11.53 (0.72)	12.93 (0.76)	12.99 (0.71)	12.01 (0.64)	11.49 (0.61)	10.29 (0.56)	11.41 (0.59)	11.16 (0.55)	-9 (-22, 7)
	FSS	7.41 (0.61)	6.63 (0.57)	13.02 (0.79)	13.8 (0.79)	9.22 (0.64)	8.91 (0.59)	7.81 (0.51)	8.09 (0.51)	8.19 (0.50)	8.58 (0.51)	7.29 (0.44)	-2 (-20, 20)
	FBS	5.31 (0.52)	6.68 (0.57)	4.99 (0.49)	3.86 (0.42)	4.24 (0.43)	3.42 (0.36)	2.27 (0.28)	3.27 (0.32)	2.89 (0.30)	2.34 (0.27)	2.85 (0.28)	-46 (-59, -30)
	OFOL	10.21 (0.72)	9.77 (0.69)	7.27 (0.59)	8.40 (0.62)	9.05 (0.63)	8.67 (0.58)	7.50 (0.50)	6.16 (0.44)	6.50 (0.45)	6.06 (0.43)	6.80 (0.43)	-33 (-45, -20)
	UF	33.55 (1.30)	30.58 (1.23)	23.42 (1.05)	21.88 (1.00)	20.97 (0.96)	17.04 (0.81)	18.33 (0.79)	15.28 (0.70)	14.78 (0.67)	17.44 (0.73)	16.65 (0.67)	-50 (-56, -45)

Notes:

1. % change in rate and its 95% CI that was calculated as “mortality rate ratio (MRR) -1”;

2. Abbreviations: FSLS: Falls on same level from slipping, tripping and stumbling (W01); FRF: Furniture related falls (W06-W08); OFSL: other falls on same level (W00, W02-W05, W09 and W18); FSS: Falls on and from stairs and steps (W10); FBS: falls from, out of or through building or structure (W13); OFOL: other falls from one level to another (W11, W12 and W14-W17); UF: unspecified falls(W19).
